# Disability weights for comorbidity and their influence on Health-adjusted Life Expectancy

**DOI:** 10.1186/1478-7954-4-1

**Published:** 2006-04-10

**Authors:** Pieter HM van Baal, Nancy Hoeymans, Rudolf T Hoogenveen, G Ardine de Wit, Gert P Westert

**Affiliations:** 1 National Institute for Public Health and the Environment, Bilthoven, The Netherlands

## Abstract

**Background:**

Comorbidity complicates estimations of health-adjusted life expectancy (HALE) using disease prevalences and disability weights from Burden of Disease studies. Usually, the exact amount of comorbidity is unknown and no disability weights are defined for comorbidity.

**Methods:**

Using data of the Dutch national burden of disease study, the effects of different methods to adjust for comorbidity on HALE calculations are estimated. The default multiplicative adjustment method to define disability weights for comorbidity is compared to HALE estimates without adjustment for comorbidity and to HALE estimates in which the amount of disability in patients with multiple diseases is solely determined by the disease that leads to most disability (the maximum adjustment method). To estimate the amount of comorbidity, independence between diseases is assumed.

**Results:**

Compared to the multiplicative adjustment method, the maximum adjustment method lowers HALE estimates by 1.2 years for males and 1.9 years for females. Compared to no adjustment, a multiplicative adjustment lowers HALE estimates by 1.0 years for males and 1.4 years for females.

**Conclusion:**

The differences in HALE caused by the different adjustment methods demonstrate that adjusting for comorbidity in HALE calculations is an important topic that needs more attention. More empirical research is needed to develop a more general theory as to how comorbidity influences disability.

## Background

Health-adjusted life expectancy (HALE) is a summary measure of population health that has been introduced as part of the Health Expectancy Network (Réseau Espérance de Vie en Santé, or REVES) and is defined as: "*a generic term for a weighted expectation of life summed over a complete set of health states*" [1]. HALE, like life expectancy, is independent of the size and composition of the population and is therefore useful to make comparisons between populations and over time [2]. One method of estimating HALE is by using data available from Burden of Disease studies [3]. As a first step to estimate HALE from Burden of Disease data, disease prevalences are coupled to disease specific disability weights to estimate the average amount of disability in a population specified by sex and age [4]. Disability weights reflect the relative severity and impact of a disease and theoretically range from 0 (no disability) to 1 (death) [5]. Then, the average amount of disability can be combined with a life table to estimate HALE. In this paper, we will focus on this specific form of HALE that has also been termed disability-adjusted life expectancy (DALE) [3,4].

Comorbidity, defined as the presence of two or more diseases in one person, complicates HALE calculations for two reasons. The first one is that the exact amount of comorbidity is unknown since all data on disease incidence, prevalence and mortality gathered in Burden of Disease (BOD) studies are disease specific [6]. The second reason is that there are no disability weights defined for comorbidity [6]. In a previous study, Barendregt and Bonneux found that HALE was generally insensitive to different methods to define disability weights for comorbidity [7]. However, they limited their research to six diseases and to comorbidity between disease pairs only. In this article, we will examine the impact of different methods to define disability weights for comorbidity on HALE estimates and compare them with HALE estimations without adjustment for comorbidity.

If burden of disease data and disability weights are used for calculations of HALE, disability weights for comorbidity are usually calculated assuming a multiplicative model [4,8]. The multiplicative model implies that disability increases with the number of conditions one has, but that the overall effect is less than additive. This is in line with findings of Verbrugge et al. [9]. They tested whether disability increased linearly with the number of chronic conditions individuals have and investigated whether there are interaction effects on disability for specific combinations of chronic diseases. They concluded that although disability increases as the number of chronic conditions increases, the marginal increase decreased as the number of conditions increases. However, they also found that in many cases the disability caused by having two diseases was not higher than having either one of the two diseases. This latter finding suggests that solely the disease that leads to most disability determines the total amount of disability in patients with multiple diseases. To investigate the effect of adjustments for comorbidity we estimated HALE using two different methods to define disability weights for comorbidity:

- multiplicative adjustment method: using this method it is assumed that the impact on disability due to comorbidity is proportional. Although disability increases with additional diseases, it is less than the sum of disability weights for the individual diseases. This is the default method used in HALE calculations [4,8];

- maximum adjustment method: using this method the disability weight for comorbidity equals the disability weight of the disease with the highest disability weight. This adjustment for comorbidity can be thought of as a maximum adjustment since having multiple diseases only leads to more disability if individual diseases lead to more disability. The total amount of disability attributed to comorbidity is equal to the highest amount of disability associated with one of the concurrent diseases.

To quantify the importance of comorbidity adjustments, we will use HALE estimates without adjustments for comorbidity as a comparator. In the next section, we describe how to estimate the average disability weight and HALE using the different adjustment methods if independence between diseases is assumed. Then, results of HALE estimates are presented. In the last section, implications of the results and directions for future research are discussed.

## Methods

In order to estimate HALE we set up an abridged life table using mortality rates for the Netherlands from 1999 [10]. The number of life years obtained from the life table were multiplied by one minus the average disability weights:



*HALE*_*g*,*a*, _*health-adjusted life expectancy gender g age a*

*L*_*g*,*a *_*number of life years lived between age a and a+5 for gender g*

*L*_*g*,85+ _*number of life years lived after age 85 for gender g*

*m*_*g*,*a *_*average disability weight between age a and a+5 for gender g*

*m*_*g*,85+ _*average disability weight after age 85+ for gender g*

*l*_*g*,*a *_*number of survivors at age a in the life table cohort for gender g*

z last open-ended age interval in the life table

Age and sex specific average disability weights are a function of disease specific prevalence rates and disability weights. In our study, data from the Dutch Burden of Disease Study was used to estimate average disability weights. The Dutch Burden of Disease Study estimated disability weights, using a large panel of experts and the person trade off method [11], and disease prevalence of 48 different disease categories [12]. All data used in our calculations (mortality rates, disease prevalences and disability weights) are available in Additional file 1.

To estimate comorbidity prevalence, independence between diseases is assumed so the amount of comorbidity between disease 1 and 2 (the joint prevalence of diseases 1 and 2) is simply the product of their prevalence rates:



*p*(*1*,*2*) *joint prevalence of disease 1 and 2*

*p*(*d*) *joint prevalence of d diseases*

Gender-specific average disability weights were calculated using age classes of five years (0–4, 5–9, 10–14 to 85+). However, for notational simplicity, age and sex indices have been omitted in the notation.

### No adjustment for comorbidity

When no adjustment for comorbidity is made the average disability weight can be calculated by simply adding up the disability caused by all diseases:



m average disability weight

*p*_*d *_*prevalence rate of disease d*

*w*_*d *_*disability weight of disease d*

Making no adjustment for comorbidity is equivalent to assuming that effects of comorbidity on disability are additive. Thus, if a person has more than one disease his total disability weight equals the sum of the disability weights for those diseases. However, in this interpretation individual disability weights may add up to more than one. This cannot be interpreted in a plausible way because it would imply that more than one year of health is lost when living for one year with those diseases.

### Multiplicative adjustment method

Using this method, it is assumed that the increase in disability due to comorbidity disability is proportional. Total disability for an individual having more diseases can be written as:



*w*(*1*,*2*) *disability weight of an individual with disease 1 and 2*

*w*(*d*) *disability weight of an individual with d diseases*

This implies that the disability due to comorbidity increases with more comorbid diseases but is less than the sum of individual disability weights for all comorbid diseases. If there are only 2 diseases the average disability weight assuming independence equals:

*m *= 1 - (1 - *p*_1_) (1 - *p*_2_) + (1 - *p*_2_) *p*_1 _(1 - *w*_1_) + (1 - *p*_1_) *p*_2 _(1 - *w*_2_) + *p*_1_*p*_2 _(1 - *w*_1_) (1 - *w*_2_) = 1 - (1 - *p*_1_*w*_1_) (1 - *p*_2_*w*_2_)     (5)

This can be generalized to *d *diseases:



### Maximum adjustment method

Compared to having one disease, having two diseases only leads to more disability if the second disease causes more disability than the first one. Assuming that the diseases are ordered in terms of disability weights, e.g. *w*_1_≥*w*_2_≥*w*_3_............*w*_*n *_someone who has disease 1 and 2 has a disability weight that equals that of *w*_1 _since disease 1 is worse than disease 2:



In order to estimate average disability weights using this method the prevalence rate for which disease *d *has the highest disability weight must be estimated (denoted *H*_*d*_). We can recursively define the prevalence rate *H*_*d *_(see Appendix for a derivation):



*H*_*d *_*prevalence rate for which disease d has the highest disability weight*

The average disability weight can then be written as:



### Comparing different methods to adjust for comorbidity

Compared to no adjustment, the multiplicative adjustment method results in a lower average disability weight but compared to the maximum adjustment method in a higher average disability weight:



For *d *diseases:



## Results

**Figure 1:**
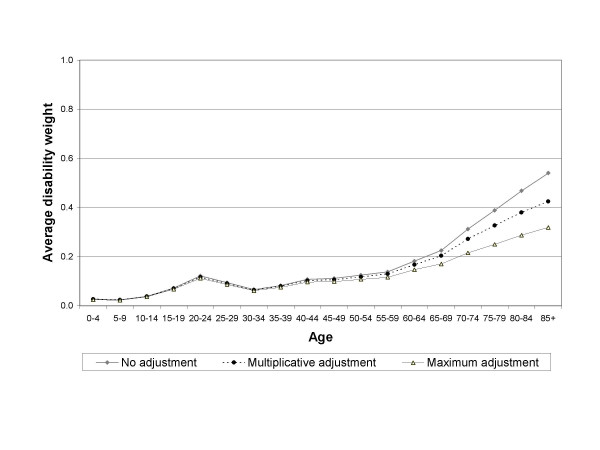
Average disability weights for men in the Netherlands 1999 using different methods to adjust for comorbidity.

**Figure 2:**
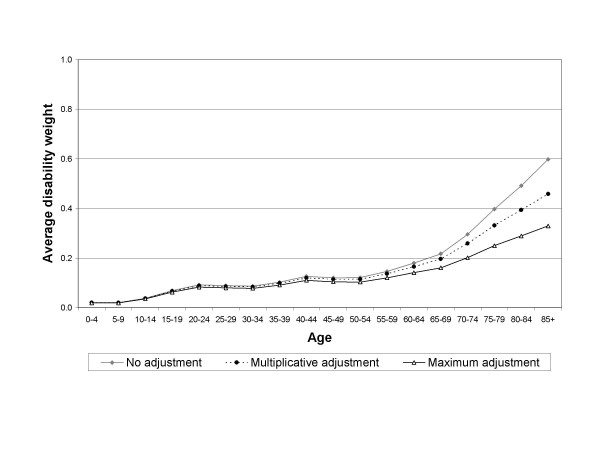
Average disability weights for women in the Netherlands 1999 using different methods to adjust for comorbidity.

Figures 1 and 2 display the estimated average disability weights for men and women, using the different adjustment methods.

By definition, the maximum adjustment method results in the lowest estimate of the average disability weight and no adjustment for comorbidity in the highest estimate. The difference between the adjustment methods increases with age. This is caused by the higher amount of comorbidity in the elderly. At age 85 and over the difference in the average disability weight between the multiplicative adjustment method and no adjustment amounts to 0.11 for men and 0.14 for women. The difference between the multiplicative and maximum adjustment methods are 0.11 and 0.13 for respectively men and women aged 85 and over. Thus, the choice for adjustment method has more implications in an elderly population than in a younger population.

Tables 1 and 2 display estimates of life expectancy and HALE. Life expectancy for Dutch males in 1999 was 75.9 years. Depending on the adjustment method for comorbidity, 65.7 to 67.9 years are considered healthy years. Females have a higher life-expectancy, and also a higher HALE. Independent of the method of adjustment, women's HALE, relative to life expectancy, is always lower than men's HALE. This reflects the fact that men more often than women die from lethal diseases with a short duration (e.g. lung cancer). At birth, the difference in HALE between the multiplicative and maximum adjustment method is 1.2 years for males and 1.9 years for females. For males, the difference in HALE between the multiplicative and maximum adjustment method declines from 1.2 years at birth to 1.0 at the age of 60. For females, the differences between the methods are somewhat larger. The difference of 1.9 years in HALE at birth between the multiplicative and maximum adjustment method declines to 1.6 at the age of 60. For both males and females at all ages, the differences between no adjustment and multiplicative adjustment are smaller than the differences between the multiplicative and maximum adjustment method.

**Table 1:** Life Expectancy (LE) and Health-adjusted Life Expectancy (HALE) for Dutch males, 1999

*Age*	**LE**	**HALE**		
		*No adjustment*	*Multiplicative adjustment method*	*Maximum adjustment method*

0	75.9	65.7	66.7	67.9
15	61.5	51.7	52.6	53.8
60	19.4	13.5	14.4	15.4

**Table 2:** Life Expectancy (LE) and Health-adjusted Life Expectancy (HALE) for Dutch females, 1999

*Age*	**LE**	**HALE**		
		*No adjustment*	*Multiplicative adjustment method*	*Maximum adjustment method*

0	81.0	68.5	69.9	71.8
15	66.6	54.4	55.8	57.6
60	23.8	15.8	17.1	18.7

## Discussion and conclusion

In this study, two different methods to adjust for comorbidity in HALE calculations were compared to HALE estimates without adjustment for comorbidity. The methods differ in the manner in which disability weights for comorbid conditions were defined. The multiplicative adjustment method implies that comorbidity increases disability but that the effects are less than the sum of disability from the individual diseases. Using the maximum adjustment method, disability is solely determined by the most severe disease. Compared to no adjustment, a multiplicative adjustment lowers HALE estimates with 1.0 years for males and 1.4 years for females. The maximum adjustment methods lowers HALE estimates compared to the multiplicative adjustment with 1.2 years for males and 1.9 years for females. Thus, the differences in HALE resulting of differences in defining disability weights are larger than the differences between no adjustment and the multiplicative adjustment method. Although we think the differences in HALE resulting of the different methods to define disability weights are important, also uncertainly related to the estimation of the prevalence of each of the 48 diseases may cause substantial variations in the average disability weights and, therefore, on HALE estimates. However, it is difficult to quantify this uncertainty because uncertainty estimates around the disease prevalence are not available [12]. In an attempt to test the sensitivity of HALE for variations in prevalence rates we calculated HALE using the multiplicative adjustment method and increased all prevalence rates with 10%. This lowered HALE estimates with less than one year for both males and females.

Contrary to our results, Barendregt and Bonneux found in a previous study that HALE was generally insensitive to different methods of weighing comorbidity [7]. However, they limited themselves to six diseases and to comorbidity between disease pairs only. In our study, all possible combinations of comorbidity between 48 diseases were investigated. The explanation for the difference in results between our study and the Barendregt and Bonneux study is straightforward: if more diseases are taken into account, there is more comorbidity, especially if comorbidity between all disease combinations is described. With a higher prevalence of comorbidity, the method used to adjust for comorbidity becomes more important.

A crucial assumption in this paper is that of independence between diseases. Although this assumption may be violated in practice, it will not influence our conclusion that adjustments for comorbidity are important in HALE calculations. In fact, assuming independence probably underestimates the amount of comorbidity since the probability of getting different diseases is not independent due to clustering of diseases as a result of genetics, biological risk factors (e.g. blood pressure, cholesterol) environmental factors (e.g. air pollution) and lifestyle (e.g. smoking and drinking) [13,14].

Different methods to define disability weights for comorbidity can have important implications not only for the estimation of HALE itself but also on applications of HALE such as monitoring trends in health or as an aid in priority setting. Even if the same methodology to adjust for comorbidity is used consistently, the choice of the adjustment method might influence the manner in which HALE reacts to trends or interventions. For instance, when using the maximum adjustment method eliminating diseases with low disability weights has smaller or even no impact on HALE compared to the multiplicative adjustment method. Furthermore, both the maximum and multiplicative adjustment methods imply that in priority setting, ceteris paribus, less priority should be given to patients with more comorbidity. For example, improving diabetes care in diabetes patients without coronary heart disease results in a larger increase in average disability than diabetes care in patients with coronary heart disease. This latter example demonstrates that the choice for comorbidity adjustment might also have implications for equity trade-offs. Moreover, for the comparison of effects of specific interventions targeted at groups with different comorbidity prevalences (e.g. young and old), the choice of adjustment method may have a different impact for different interventions, and thus may affect the conclusion.

The problems caused by comorbidity in HALE calculations are not present if self reported generic measures of health such as the EQ-5D are used. [15-17] Then, patients decide for themselves how the total disability that is caused by all concurrent diseases influences their functioning and quality of life. Therefore, empirical results of studies using self reported generic measures could be used to develop a more general theory as to how comorbidity influences disability. For instance, a question that needs to be answered is whether two comorbid diseases from different "disease clusters" (such as a physically limiting disease combined with a mental disease) leads to more disability than two or more diseases from one "disease cluster". Such a theory might be used to justify the choice for a specific adjustment method in HALE calculations.

## Competing interests

The author(s) declare that they have no competing interests.

## Authors' contributions

PHMvB carried out the analyses and drafted the manuscript. RTH contributed to the mathematics. NH provided the data. All authors contributed to the writing of the paper.

## Appendix: derivation of average disability weights for maximum adjustment method

The fraction of the population that gets the weight of disease *d *equals the prevalence rate of disease *d *minus the fraction of the population that has disease *d *but also a disease with a higher disability weight. Assuming that the diseases are ordered in terms of disability weights, e.g. *w*_1_≥*w*_2_≥*w*_3 _.....................*w*_*n *_we can write this principle for three diseases as:

*H*_1 _= *p*_1_

*H*_2 _= *p*_2 _*(1 - *p*_1_)

*H*_3 _= *p*_3 _*(1 - (*p*_1 _+ *p*_2 _- *p*_2 _* *p*_2_))

*H*_*d *_*prevalence rate for which disease d has the highest disability weight*

*p*_*d *_*prevalence rate of disease d*

This can be rewritten to:

*H*_1 _= *p*_1_

*H*_2 _= (1 - *H*_1_) *p*_2_

*H*_3 _= *(1 - H*_1 _- *H*_2_) *p*_3_

Using this we can recursively define the prevalence rate *H*_*d*_:



The average disability weight can then be written as:



m average disability weight

*w*_*d *_*disability weight of disease d*

## Supplementary Material

Additional file 1 Click here for file
